# Influence of Pokémon Go on Physical Activity: Study and Implications

**DOI:** 10.2196/jmir.6759

**Published:** 2016-12-06

**Authors:** Tim Althoff, Ryen W White, Eric Horvitz

**Affiliations:** ^1^ Computer Science Department Stanford University Stanford, CA United States; ^2^ Microsoft Research Redmond, WA United States

**Keywords:** physical activity, Pokémon Go, mobile health, mHealth, wearable devices, mobile applications, games, exergames, public health

## Abstract

**Background:**

Physical activity helps people maintain a healthy weight and reduces the risk for several chronic diseases. Although this knowledge is widely recognized, adults and children in many countries around the world do not get recommended amounts of physical activity. Although many interventions are found to be ineffective at increasing physical activity or reaching inactive populations, there have been anecdotal reports of increased physical activity due to novel mobile games that embed game play in the physical world. The most recent and salient example of such a game is Pokémon Go, which has reportedly reached tens of millions of users in the United States and worldwide.

**Objective:**

The objective of this study was to quantify the impact of Pokémon Go on physical activity.

**Methods:**

We study the effect of Pokémon Go on physical activity through a combination of signals from large-scale corpora of wearable sensor data and search engine logs for 32,000 Microsoft Band users over a period of 3 months. Pokémon Go players are identified through search engine queries and physical activity is measured through accelerometers.

**Results:**

We find that Pokémon Go leads to significant increases in physical activity over a period of 30 days, with particularly engaged users (ie, those making multiple search queries for details about game usage) increasing their activity by 1473 steps a day on average, a more than 25% increase compared with their prior activity level (*P*<.001). In the short time span of the study, we estimate that Pokémon Go has added a total of 144 billion steps to US physical activity. Furthermore, Pokémon Go has been able to increase physical activity across men and women of all ages, weight status, and prior activity levels showing this form of game leads to increases in physical activity with significant implications for public health. In particular, we find that Pokémon Go is able to reach low activity populations, whereas all 4 leading mobile health apps studied in this work largely draw from an already very active population.

**Conclusions:**

Mobile apps combining game play with physical activity lead to substantial short-term activity increases and, in contrast to many existing interventions and mobile health apps, have the potential to reach activity-poor populations. Future studies are needed to investigate potential long-term effects of these applications.

## Introduction

“Those who think they have no time for bodily exercise will sooner or later have to find time for illness.” [Edward Stanley, Earl of Derby, December 20, 1873]

Physical activity is critical to human health. People who are physically active tend to live longer, have lower risk for heart disease, stroke, type 2 diabetes, depression, and some cancers, and are more likely to maintain a healthy weight [1,2,3,4]. Recent analyses estimate that physical inactivity contributes to 5.3 million deaths per year worldwide [5] and that it is responsible for a worldwide economic burden of US $67.5 billion due to health care expenditures and productivity losses [6]. Only 21% of US adults meet official physical activity guidelines [7,8] (at least 150 minutes a week of physical activity for adults), and less than 30% of US high school students get at least 60 minutes of physical activity every day [7]. Efforts to stimulate physical activity hold opportunity for improving public health. Numerous studies have called for population-wide approaches [9,10]. However, many interventions have been found to be either ineffective [11,12] in reaching only populations that were already active [13,14] or not scalable across varying cultural, geographic, social, and economic contexts [9].

Of late, there have been anecdotal reports of novel mobile games leading to increased physical activity, most notably for Pokémon Go [15] (other examples include Ingress and Zombies, Run!). Pokémon Go is a mobile game combining the Pokémon world through augmented reality with the real world requiring players to physically move around. Pokémon Go was released in the United States on July 6, 2016 and was adopted widely around the world—25 million active users in the United States [16] and 40 million worldwide [17]; 500 million downloads worldwide [18]. Due to this massive penetration, Pokémon Go can be viewed as an intervention for physical activity on a large scale. However, its effectiveness for stimulating additional walking is yet to be determined.

Our study seeks to provide guidance on large-scale interventions represented by the Pokémon Go phenomenon and on the possibilities for increasing physical activity which could be leveraged games for public health purposes. We study the influence of Pokémon Go on physical activity through a combination of wearable sensor data and search engine query logs for 31,793 users over a period of 3 months. Within these users, we identify 1420 Pokémon Go users based on their search activity and measure the effect of playing the game on their physical activity. We compare changes in physical activity for Pokémon Go users with changes for a large control group of US wearable users and further compare the effect of Pokémon Go with 4 leading mobile health apps. Finally, we estimate the impact of Pokémon Go on public health.

To summarize, our main research questions are

1. Is playing Pokémon Go associated with increases in physical activity? How large is this effect and how long does it persist?

2. Is this effect restricted to particular subpopulations or is it affecting people of all prior activity levels, ages, gender, and weight status?

3. How does Pokémon Go compare with leading mobile health apps in terms of its ability to change physical activity?

4. How has Pokémon Go affected physical activity in the United States and what is its potential impact on public if the game was able to sustain the engagement of its users?

To the best of our knowledge, this is the first study to combine large-scale wearable and search sensors to retrospectively evaluate large-scale interventions and the first to study the effect of Pokémon Go.

## Methods

### Dataset

We combine data from search engine queries with physical activity measurements from wearable devices. Specifically, we jointly analyze (1) queries to the Microsoft Bing search engine mentioning “pokemon.” We use this to identify which users are likely playing Pokémon Go; and (2) physical activity as measured through daily number of steps on the Microsoft Band. We jointly use these data to measure differences in physical activity before and after each user shows strong evidence of starting to play Pokémon Go.

The main study population is the 31,793 US users of Microsoft products who have agreed to link data from their Microsoft Band wearables and their online activities to understand product usage and improve Microsoft products. We show that 1420 users can be classified as Pokémon Go players with high confidence. We compare changes in physical activity in this population with changes in a control group consisting of a random sample of 50,000 US Microsoft Band users. As the expected fraction of Pokémon Go players in this random sample is very low [19], we are confident that this does not affect our analyses. For all users, we have self-reported age, gender, height, and weight, which are used to estimate the effect of Pokémon Go on different groups of users.

This research was conducted while all authors were employed by Microsoft. Our work was conducted offline, on data collected to support existing business operations, and did not influence the user experience. All data were anonymized prior to analyses. The Ethics Advisory Committee at Microsoft Research considers these precautions sufficient for triggering the Common Rule, exempting this work from detailed ethics review.

**Table 1:** Representative experiential and nonexperiential Pokémon Go queries [20]. In total, 454 such features were used for classification of experiential queries.

Nonexperiential query	Experiential query
pokemon go	pokemon go iv^a^ calculator
pokemon go death san francisco	pokemon go teams
pokemon go robberies	how to play pokemon go
couple sues pokemon go	pokemon go guide
baltimore pokemon accident	pokemon go servers
pokemon games	pokemon go bot
bluestacks^b^ pokemon go	pokemon go eevee^c^ evolution

^a^iv: It refers to individual values which are attribute points of Pokémon determining their stamina, attach, and strength.

^b^bluestacks: It refers to a method to play Pokémon Go on a desktop computer instead of the intended use in the real world.

^c^eeevee: It is the name of a Pokémon.

### Identification of Pokémon Go Users Through Search Queries

We collected all Bing search queries from any device (eg, desktop, laptop, or mobile) of the 31,793 users between July 6, 2016 (US release date of Pokémon Go) and August 23, 2016 (date of statistical analysis) that mention the term “pokemon” (ignoring capitalization). We then manually annotated the 454 most frequent unique queries in terms of whether they are experiential [20,21]; that is, the user is very likely playing Pokémon Go, rather than just being interested in it for some other reason such as following up on news reports or general interest in the game. This was done by an author familiar with the game manually executing each query and judging whether the query and search engine results provided compelling evidence of someone playing the game. Examples for experiential and non-experiential queries are given in Table 1.

Among the 25,446 users who issued any queries during our time of observation, 1420 or 5.58% issued an experiential query for Pokémon Go. This number closely matches the estimated fraction of regular Pokémon users in the United States—5.9% according to [19]—suggesting that our search-engine based method is effectively detecting a large number of Pokémon Go users. We use the time of each user’s first experiential query for Pokémon Go as a proxy for the time when they started playing Pokémon Go and denote this time as *t*_0_.

Note that our method of identifying Pokémon Go players through experiential queries can potentially overestimate *t*_0_, if players perform these queries several days after starting to play the game, but the opposite is less likely due to the nature of experiential queries targeting specific aspects of game play (see Table 1). However, note that any potential overestimates of *t*_0_ lead to more conservative estimates of the effect of Pokémon Go as potential game-related increases in activity would be counted as activity before *t*_0_ (assuming the effect is non-negative).

### Measuring Physical Activity

We seek to measure the change in physical activity before and after the time of the first experiential query for Pokémon Go, *t*_0_, when a user presumably started playing the game. We measure physical activity through daily steps as recorded by the 3 axis accelerometer and gyrometer of a wrist-worn consumer activity tracking ftdevice (Microsoft Band). Accelero­meter-defined activity measures are preferred over subjective survey-based methods, which have been found to overestimate physical activity by up to 700% [22]. We use steps data from 30 days before the first experiential query (*t*_0_) until 30 days after the first experiential query. We note that, at the time of this study, very few users had been using Pokémon Go for more than 30 days. Furthermore, note that all Pokémon Go users included in our dataset have been using the wearable device for a significant amount of time (median 433 days) such that differences in activity cannot be due to starting to use the wearable device. As not every search engine user whom we identified as a Pokémon Go player is also regularly tracking steps, there are 792 users who tracked steps on at least one day before and after *t*_0_ (see Table 2). We restrict our analysis to these users and compare their activity with the control group described below. Note that the choice of threshold (number of days tracked before and after *t*_0_) does not significantly affect our analysis, as we find very similar results when restricting our analysis to users tracking, for example, 7 days before and after *t*_0_.

**Table 2:** Number of Pokémon Go users and number of days of steps tracking for these users included in the dataset. We counted days up to 30 days before and after each user’s first experiential query and only considered users with at least one day tracked before and after their first experiential query.

Minimum number of experiential Pokémon Go queries	Number of users	Number of days with steps data
1	792	36,141
2	417	18,804
3	262	11,916
4	199	9132
5	143	6633
6	113	5186
7	85	3819
8	70	3131
9	56	2512
10	50	2218

#### Control Group

We further compare the differences in activity in the Pokémon Go user population with any changes in the control group, a random sample of US Microsoft wearable users. For example, summertime along with improved weather conditions and potential vacation time might be linked to increases in the steps of the control group as well. As there are no experiential queries for any of the control users, we need to define a suitable substitute for *t*_0_ for the control group in order to compare both groups. We use this reference point *t*_0_ to measure changes in physical activity before and after for both the Pokémon Go user group as well as control users. For the Pokémon Go users, *t*_0_ corresponds to the date of the first experiential query for Pokémon Go (eg, July 6, 2016 or July 7, 2016). One could consider using a single point in time *t*_0_ for all control users, for example the July 6, 2016 release date of Pokémon Go. However, this choice would temporarily align all control users such that weekend, weather, or other effects could lead to confounding. In the Pokémon Go user group, all users have potentially different *t*_0_ based on their first experiential query and therefore such effects are not aligned. In order to match observation periods between both groups, we therefore use the exact same distribution of *t*_0_ for control users; that is, for each control user, we randomly sample a Pokémon Go user and use the same value for *t*_0_ for the control user. This ensures that we will compare physical activity over matching observation periods.

#### Wear Time

We also measure the wear time of the activity tracking device for each day in the dataset as the number of half hours in a day during which the device was worn on the wrist. Differences in recorded number of steps could potentially stem from simply an increase in wear time rather than an actual increase of physical activity. However, we find that during the study duration the wear time for both Pokémon Go and control users was effectively constant with the ratio between the groups changing by <1% over time. Therefore, we attribute any differences in recorded number of steps to an actual increase in physical activity due to the engagement with Pokémon Go.

### Measuring the Impact of Pokémon Go

We measured the impact of Pokémon Go on physical activity by comparing activity levels before and after each user’s first experiential query and relate the level of engagement measured through search queries to the size of the effect on physical activity.

#### Longitudinal Analysis

We compared the physical activity levels of Pokémon Go users with those of the control group population over time in relation to every user’s first experiential query (*t*_0_). As described above, we used randomly sampled *t*_0_ for users in the control group. We measured the average number of steps over a period of 30 days before the first experiential query until 30 days after the first experiential query. Note that on some days a user might not have recorded any steps and we ignore this user on that day. We measured this average activity separately for the Pokémon Go user group and the control group. To improve graph readability, we smoothed the daily average activity through Gaussian-weighted averaging with a window size of 7 days, but we reported statistical tests on the raw data. We estimated 95% CIs through a bootstrap with 500 resamples [23].

#### Dose-Response Relationship Between Pokémon Go and Physical Activity

Dose-response relationships between the amount of physical activity and various health outcomes have been well established [4,24,25]. We expect that high engagement with Pokémon Go would be reflected in a larger number of experiential queries. Particularly engaged users might also exhibit larger increases in physical activity. We quantified the exact effect sizes for these increases and studied this potential dose-response relationship between the Pokémon Go–related engagement on a search engine and real-world physical activity. We measured the difference in the average number of daily steps across all users and days for the 30 days before versus 30 days after each user’s first experiential query as the effect size.

#### Does Everyone Benefit?

We measured the effect on individual user’s physical activity after starting to play Pokémon Go and related the magnitude of this effect to demographic attributes of the user including age, gender, weight status (body mass index, BMI), and prior activity level. We investigated whether only certain user groups are benefiting from the game or whether the potential health benefits might apply more widely to the game’s user population. We estimated the effect of playing Pokémon Go on each individual user defined as the difference in the average number of daily steps 30 days before and 30 days after the first experiential query. As this analysis was on user-level, we included only Pokémon Go users with at least seven days of steps tracking before and after this event to reduce noise and applied the same requirement to the control group (we used a threshold of 1 day for analyses on day-level). These constraints resulted in 677 Pokémon Go users and 26,334 control users.

#### Comparison With Existing Health Apps

We compared the effect of Pokémon Go with the effect of other mobile health apps. The Microsoft Band can be connected to other fitness and health apps and we have data on when these connections first happen (ie, explicit knowledge of *t*_0_ for users of these apps). We studied 4 leading mobile health apps with anonymized names for legal reasons. These apps are regularly rated among the top health apps on both iOS and Android platforms and represent the state-of-the-art in consumer health apps. We also measured the number of daily steps 30 days before a user starts using one of these apps until 30 days after. We only included users who started using the health apps after July 1, 2016 to control for seasonal effects and make the data comparable with our Pokémon Go user group. Again, we restricted our analysis to users who were tracking steps on at least seven days before and after the first experiential query (for Pokémon Go group) or first connecting the health app (for the comparison groups). For the 4 apps, 1155 users were included for app A, 313 for app B, 625 for app C, and 296 users for app D. Note that these users had been using the wearable device for a significant amount of time before connecting to the health app (median time in days for the 4 apps are 87, 57, 103, and 76 days, respectively). Therefore, any differences in average activity were likely due to the connected health app rather than cumulative effects of starting to use a wearable activity tracker. We did not have access to data on the level of engagements with the app.

#### Estimating the Public Health Impact of Pokémon Go

In order to quantify the effect of Pokémon Go on public health, we estimated (1) how many steps were added to US users’ physical activity during the first 30 days, (2) how many users met physical activity guidelines before and after Pokémon Go, and (3) the potential impact on life expectancy if Pokémon Go could sustain the engagement of its users.

The official physical activity guidelines [7,8] are equivalent to approximately 8000 daily steps [26,27]. Only 21% of US adults meet these guidelines. We use all users tracking steps at least seven days before and after their first experiential query for Pokémon Go. We then measured the fraction of users with more than 8000 average daily steps both 30 days before and after the first experiential query. This analysis was repeated for Pokémon Go users with at least one and at least ten experiential queries, and the control group.

If there is a substantial impact on physical activity, Pokémon Go could have a measurable impact on US life expectancy due to well-established health benefits of physical activity on heart disease, stroke, type 2 diabetes, depression, some cancers, obesity, and mortality risk [1,2,3,4,5,6]. If we assume that Pokémon Go users would be able to sustain an activity increase of 1000 daily steps, this would be associated with a 6% lower mortality risk. Using life-table analysis similar to [5] based on mortality risk estimates from [28] and the US 2013 Period Life Table [29], we estimated the impact on life expectancy based on this reduction of mortality risk.

## Results

### Study Population Demographics

Demographic statistics on identified Pokémon Go users and control users are displayed in Table 3. We found that Pokémon Go users are younger than the average user in our wearable dataset, and much less often female. The gender bias is at least in part due to the gender bias in what users agreed to link data from their Microsoft Band wearables and their online activities to understand product usage and improve Microsoft products. However, there is no evidence for a significantly different effect between males and females in our dataset. Furthermore, there is a significant fraction of overweight and obese users, similar to the proportion expected in the US population [30]. This fraction of overweight and obese users is very similar in the Pokémon Go and control user groups indicating lack of a selection effect based on weight status. The average activity level of Pokémon Go users is below that of the control group indicating that that Pokémon Go is attracting users that get less than average activity. Note that this difference is unlikely to stem from other differences between the 2 groups as younger users are typically more active than older users and males typically get more physical activity than females [12] (ie, we would expect a larger number of steps for the Pokémon Go group given the other differences). Therefore, we do not match the 2 groups on demographic attributes. However, we studied how the effects vary across demographics (see the section “Does Everyone Benefit?”).

**Table 3:** Dataset statistics.

Dataset statistic	Pokémon Go users	Wearable users^b^
Number of users	1420	50,000
Number of users with sufficient activity data	792	26,334
Median age	33	42
% female	3.8	25.7
% underweight (BMI^a^<18.5)	1.1	1.2
% normal weight (18.5≤BMI<25)	34.2	31.4
% overweight (25≤BMI < 30)	36.5	38.4
% obese (30≤BMI)	28.2	29.1
Average daily steps overall	6258	6435

^a^BMI: Body Mass Index

^b^Wearable users refers to random sample of US Microsoft Band users. We only consider users with at least one day of steps tracking before and after the user’s first experiential query (“sufficient activity data”).

**Figure 1:**
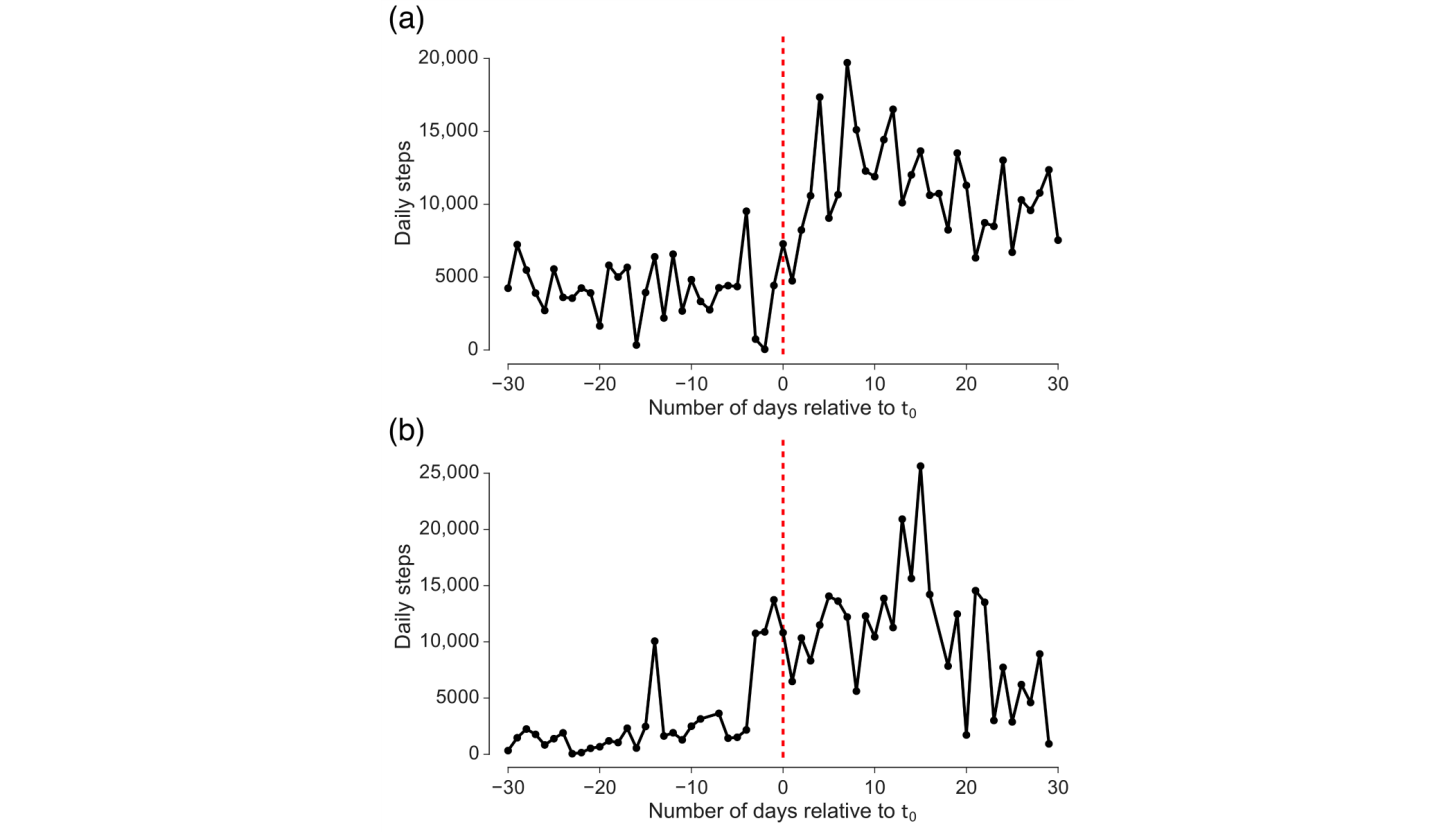
Time series of daily steps for 2 sample users (a, b). Both cases show significant increases in daily steps after the first experiential query for Pokémon Go (*t*_0_). Whereas before *t*_0_ both users take less than 5000 steps a day, after *t*_0_ they regularly reach around 15,000 steps a day.

### Example Time Series of Physical Activity

Figure 1 displays the daily number of steps before and after the user’s first experiential query for 2 example users. Both users significantly increased their activity after their first experiential query for Pokémon Go by several thousand steps each day. We analyzed whether this large increase in physical activity is representative of the study population and how it varied across individuals.

### Longitudinal Analysis

**Figure 2:**
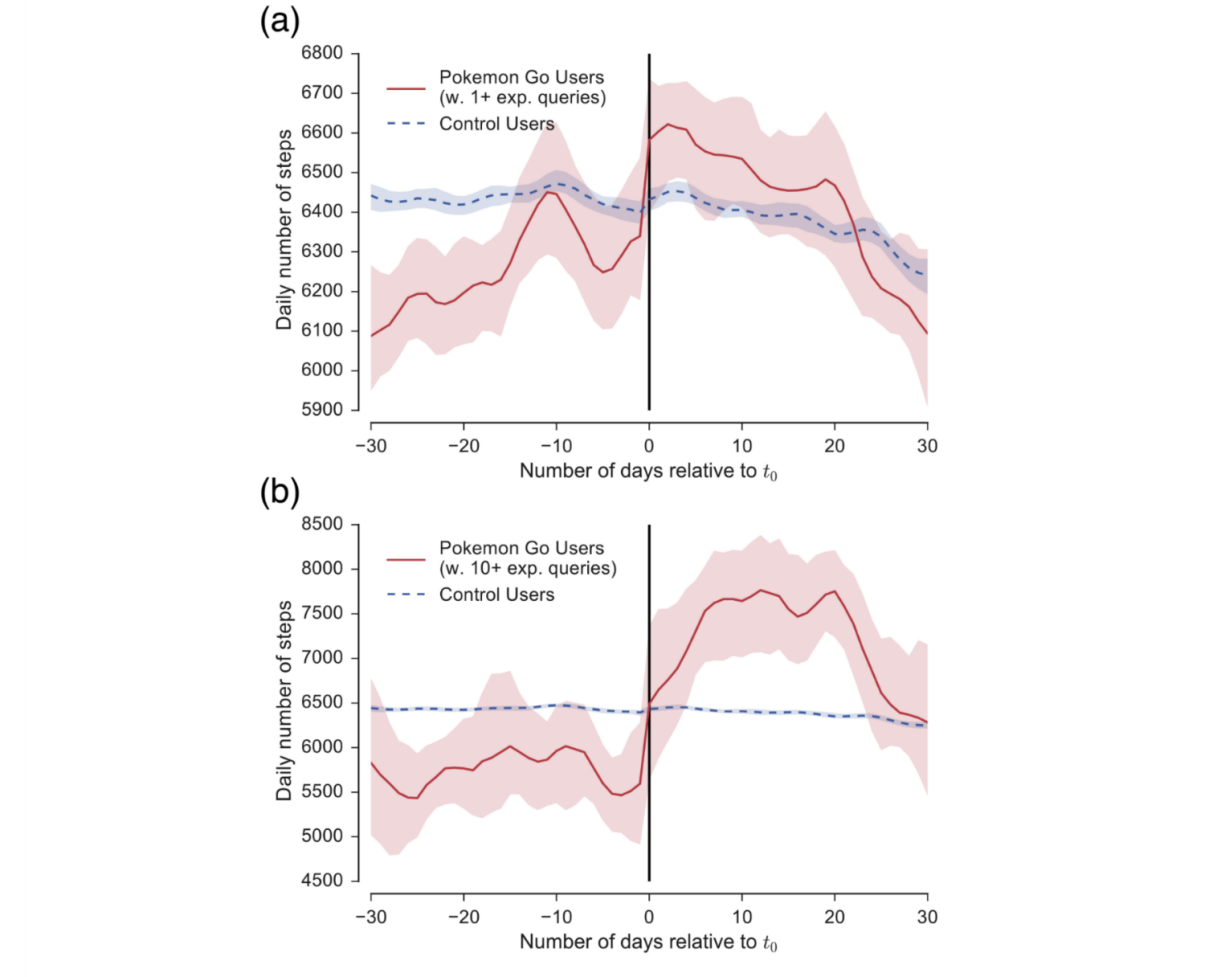
Effect of Pokémon Go on physical activity. Plots show daily steps in absolute numbers for both Pokémon Go users (red) and control users (blue). Plot (a) shows effect for users with at least one experiential query. Plot (b) shows effect for users with at least ten experiential queries. In particular for the users who show significant interest in Pokémon Go (b), we observe large average increases of 1473 steps or 26% over the 30 days following on the first experiential query. Over the same time, the control group (same for both plots) decreased their activity by 50 daily steps on average. Error bars (shaded) in this and all following plots correspond to bootstrapped 95% CIs [23].

Starting to play Pokémon Go is associated with significant increases in physical activity (Figure 2) compared with the control group. The control group slightly decreased their activity by 50 daily steps on average (*P*<.001; we use Mann–Whitney U-Tests for hypothesis tests unless noted otherwise). In contrast, Pokémon Go users increased their activity by 192 daily steps (*P*<.001). The plot shows a steep increase immediately after the first experiential query (*t*_0_), suggesting that the observed increased activity indeed stemmed from engaging with Pokémon Go (*P*<.01, comparing 3 days before and after *t*_0_). We found that Pokémon Go users initially had less activity than the average Microsoft Band user in the United States (dashed blue line; 178 daily steps less; *P*<.001). However, following the start of Pokémon Go play, their activity increased to a level larger than the control group (65 daily steps more; *P*<.001). The activity increase 10 days before *t*_0_ seemed to be statistical noise as we found no evidence for this period to stem from weekend activity or the beginning of summer vacation time due to the variation of *t*_0_ across users.

Activity increases were larger for Pokémon Go users with at least ten experiential queries; that is, users who showed significant interest in Pokémon Go (Figure 2; bottom row). These users were initially significantly less active than the average Microsoft Band user in the United States, getting 5756 daily steps compared with 6435 daily steps in the control group (*P*<.001). After they started playing Pokémon Go, they exhibited a large increase in activity to an average of 7229 daily steps (1473 daily steps difference; *P*<.001), which is about 13% larger than the control population (*P*<.001). This observation suggests that there is a dose-response relationship between interest in Pokémon Go and the effect on physical activity, which we analyze next.

It should be noted that increases in steps before *t*_0_ could stem from starts with the game in advance of queries about Pokémon Go, as we were using the first experiential query as a proxy for the start of play. If users begin to play without ever issuing a search query about Pokémon Go, we could see increases in activity before *t*_0_. However, as we observed steep increases in activity exactly at *t*_0_, this suggests that the proxy for starting is valid for most users.

Note that physical activity for both Pokémon Go user groups (top and bottom rows) decreased again after about 3 to 4 weeks after the first experiential query. However, also note that the activity for the more strongly engaged group (bottom) dropped down to a higher level than they started out with. This suggests that there could be a longer-term behavior change and that future work is needed to study long-term effects of Pokémon Go.

**Figure 3:**
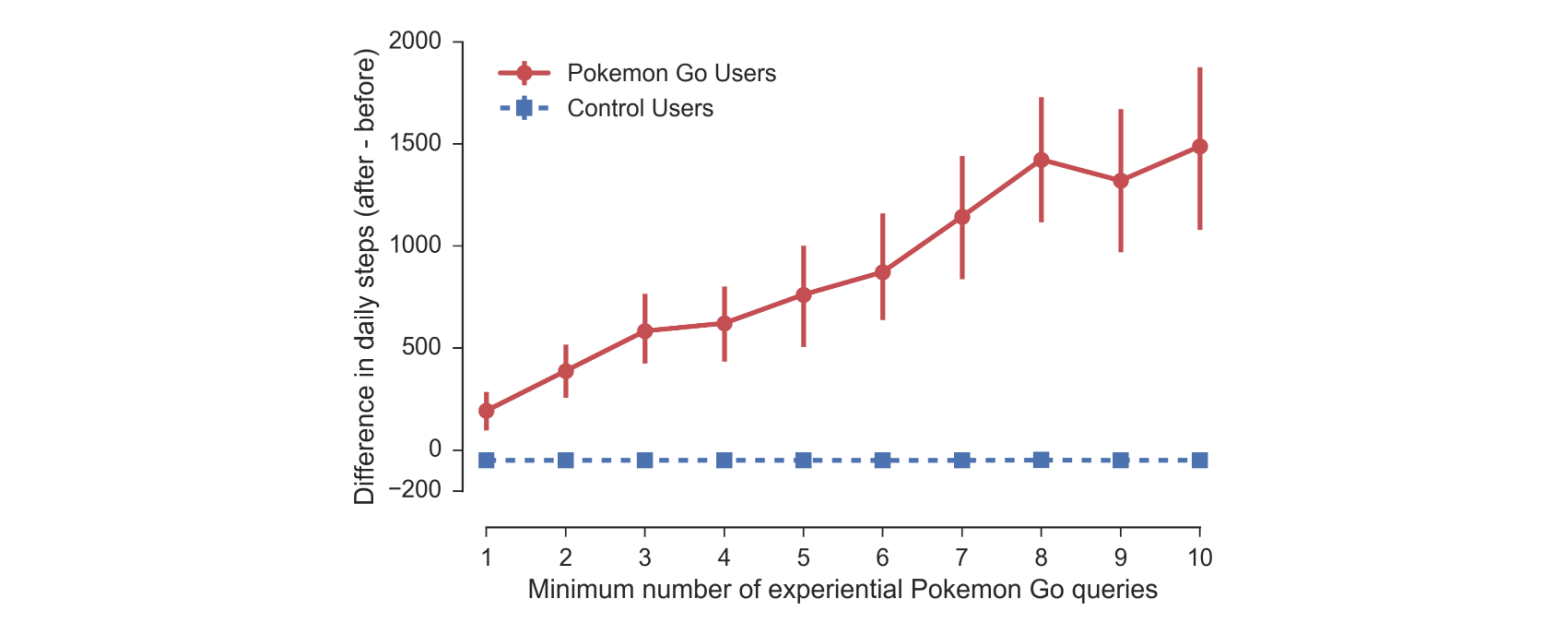
Effect sizes measuring the difference in average number of daily steps between the periods before and after *t*_0_, for different user populations based on the minimum number of experiential queries; that is, toward the right the group of users becomes smaller and increasingly interested in Pokémon Go. At any level, there are significant differences between the effect for Pokémon Go users (red) and the control users (blue). The effect increases linearly with the number of Pokémon Go queries. This dose-response relationship between expressed interest in Pokémon Go and physical activity suggests that these users are in fact playing Pokémon Go and that playing the game makes them more active. CIs for the control group are too small to be visible.

### Dose-Response Relationship Between Pokémon Go and Physical Activity

We found a dose-response relationship between the level of engagement with Pokémon Go and the effect on physical activity; that is, users with more experiential queries for Pokémon Go exhibited larger increases in physical activity (see Figure 3). For users who expressed any interest in Pokémon Go, we found significant increases in activity compared with the control group which decreased their activity by 50 steps a day. 

Furthermore, we found that these increases in steps scale roughly linearly with the number of experiential queries from 192 daily steps increase (3%) for users with 1 or more experiential queries up to an increase of 1473 daily steps (26%) for users with 10 or more experiential queries. Furthermore, the linear increase in physical activity with the number of experiential Pokémon Go queries suggests that activity increases observed in users querying a search engine for Pokémon Go are causally explained by their engagement with Pokémon Go. If there were other confounding factors that explained the difference in activity between our Pokémon Go group and the control group over time and those changes had nothing to do with Pokémon Go, then one would not expect to find such a clear dose-response relationship as given in Figure 3.

### Does Everyone Benefit?

**Figure 4:**
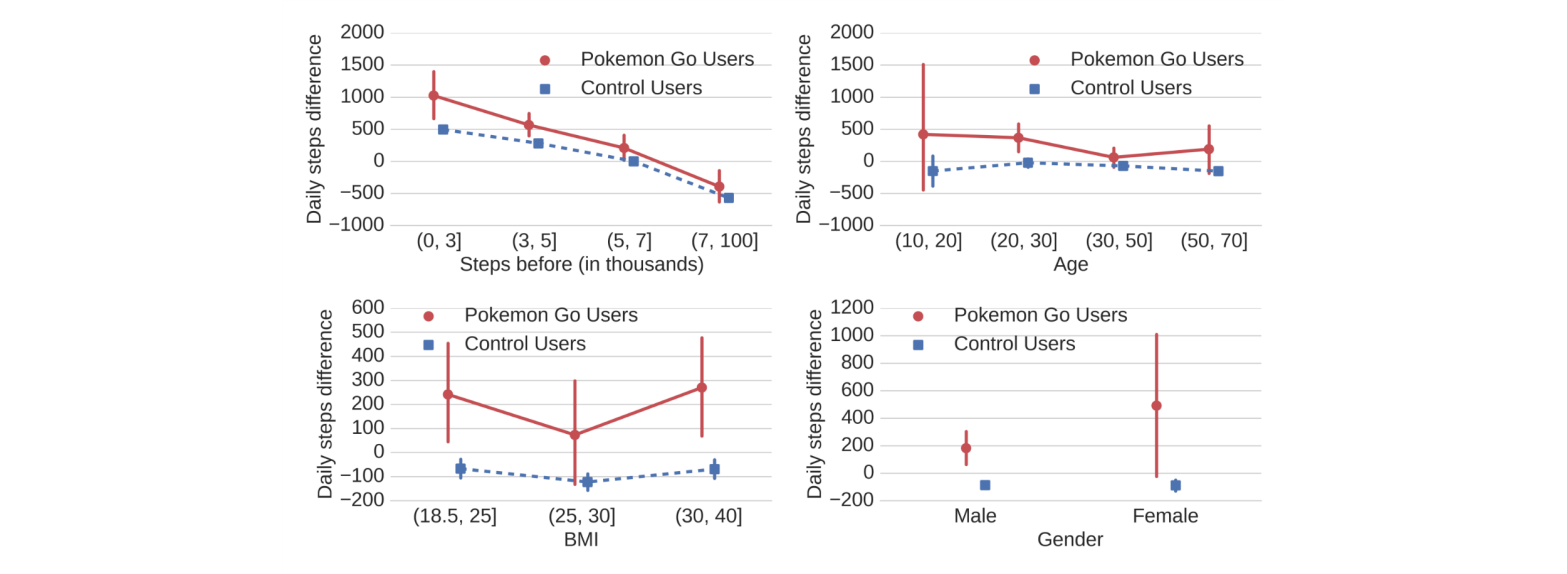
Effect sizes of physical activity increase or decrease by user demographics, including prior physical activity level (top left), age (top right), body mass index (BMI; bottom left), and gender (bottom right). In all cases, we find that Pokémon Go users (red) exhibit larger changes than their respective control group (blue; see Methods). These results suggest that physical activity increases due to Pokémon Go are not restricted to particular subgroups of users but widely spread across the overall study population.

We found that Pokémon Go increased physical activity across men and women of all ages, BMI levels, and prior activity levels (Figure 4). In particular, we found that both Pokémon Go users and control users who were very inactive exhibited large activity increases and users who were relatively active exhibited a decrease in activity on average. However, we found that Pokémon Go users exhibited larger effects than the control across all levels of prior activity (all *P*<.025). We found the largest differences between the 2 groups of users who previously were sedentary, that is, below 5000 daily steps [31]. Furthermore, Pokémon Go users exhibited bigger increases in activity than control users across all age groups (all *P*<.040; except 10-20 year old group which was small), though we found the largest effects for younger users between 10 and 30 years. We also found that the positive effect on physical activity did not vary much across all BMI groups, which is encouraging as obese individuals (30<BMI≤40) were typically less active than healthy subjects [32]. The activity differences in the Pokémon Go groups were always larger than the differences in the control group across all BMI groups (all *P*<.021). Finally, we found that activity differences in the Pokémon Go groups were larger than the differences in the control group for both men and women (all *P*<.022). Increases for women were not significantly different from increases for men (*P*=.110; note small sample size for women).

**Figure 5:**
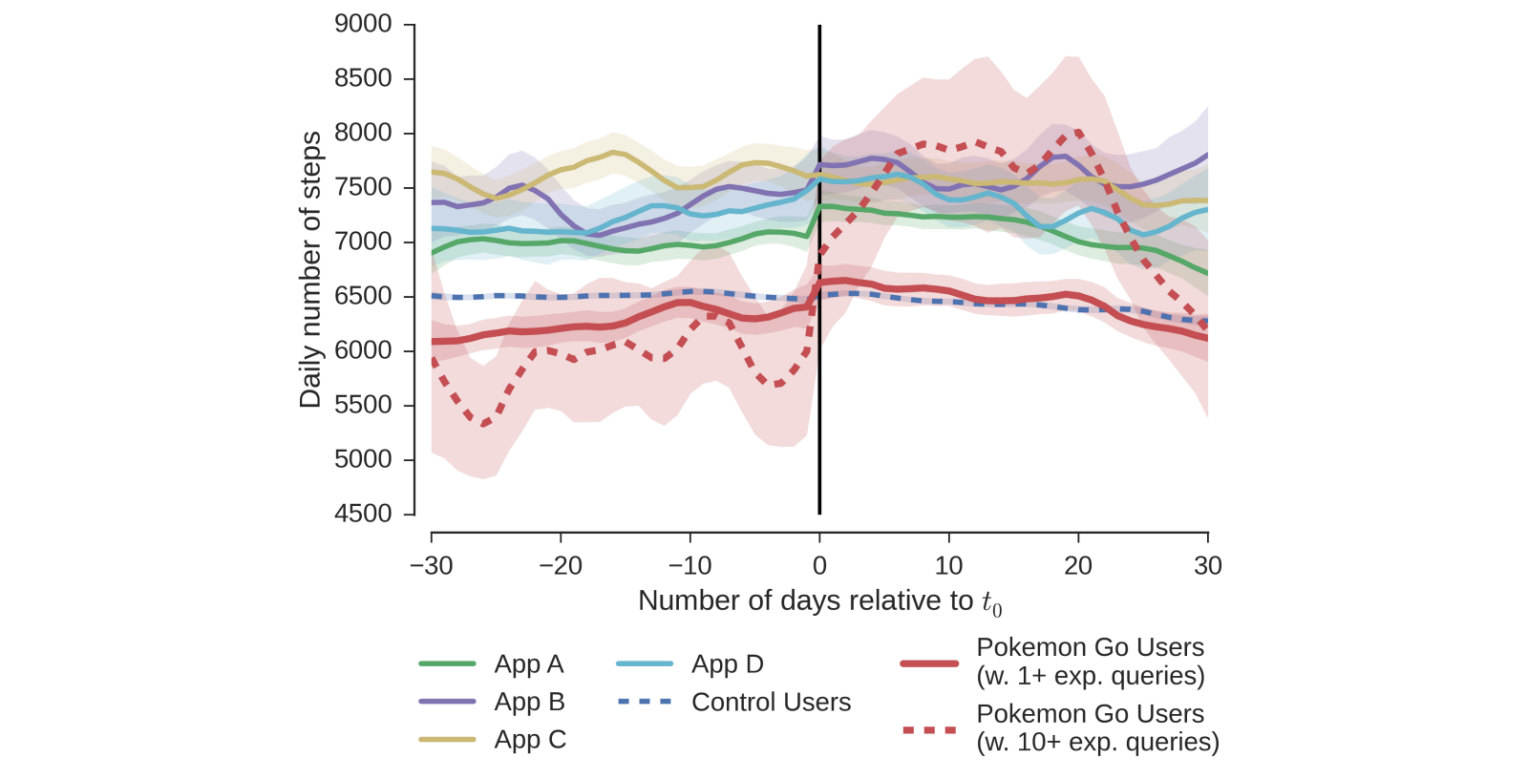
Comparing the effect of the Pokémon Go app with leading consumer health apps (A, B, C, and D). Pokémon Go users are less active than the average wearable user (control) before starting to play, but see larger increases in physical activity compared with the 4 consumer health apps.

### Comparison With Existing Health Apps

Pokémon Go led to larger increases in physical activity than other mobile health apps and further attracts more users who are not yet very active. The average daily steps over time is visualized in Figure 5 (using same smoothing method as before). First, we observed that users of all 4 health apps were significantly more active than the average wearable user (6514 daily steps) even before they started to use the health app (6997-7616 daily steps; see activity before t_0_ in Figure 5 for apps A, B, C, and D). In contrast, Pokémon Go users were less active than the average user (5901-6265 daily steps). The temporal pattern for the health apps did not contribute strong evidence that these apps were leading to significant behavior change. One exception was app A with its users significantly increasing their activity at day 0. However, this increase in activity was lower compared with the effect of Pokémon Go. Users of app A increased their activity on average by 111 daily steps or 1.6%. Compare this with 194 (1502) daily steps or 3.1 (25.5%) for Pokémon Go users with at least 1 (10) experiential queries. In particular, users demonstrating large engagement with Pokémon Go exhibited much larger increases in activity than users of any other app in our comparison.

### Estimating the Public Health Impact of Pokémon Go

#### Effect on US Physical Activity

On average, users with one or more experiential query for Pokémon Go increased their physical activity by 192 steps a day for the next 30 days (see results on dose-response relationship). Extrapolating this average effect size to 25 million Pokémon Go users in the United States [16], we found that Pokémon Go added 144 billion steps within the first 30 days to US physical activity. This is equivalent to walking around the equator 2724 times or 143 round trips to the moon.

#### Effect on Meeting Activity Guidelines

Using all users tracking steps at least seven days before and after their first experiential query for Pokémon Go, we found that that the fraction of users meeting physical activity guidelines, that is, getting 8000 average daily steps [26,27] stays approximately constant for users with one or more experiential queries (22.0% before vs 21.9% after *t*_0_) and control users (24.1% before vs 23.5%). Note that these estimates are consistent with published estimates in the US adult population (21%) [7,8]. However, for highly engaged Pokémon Go users with at least ten experiential queries, we found that during the 30 days after they started playing, 160% more users achieved 8000 average daily steps (12.2% before vs 31.7% after; relative increase of 160%). For comparison, 21% of US adults met these guidelines [7,8].

#### Effect on Life Expectancy

We found that more engaged users exhibited average physical activity increases of up to 1473 daily steps. This substantial impact on exercise across the society could have a measurable impact on US life expectancy due to well-established health benefits of physical activity [1,2,3,4,5,6]. If we assume that Pokémon Go users, between 15 and 49 years old, would be able to sustain an activity increase of 1000 daily steps, this would be associated with 41.4 days of additional life expectancy. Across the 25 million US Pokémon Go users [16], this would translate to 2.825 million years additional life added to US users. However, future studies are needed to investigate the long-term effect of Pokémon Go.

## Discussion

### Principal Findings

The Pokémon Go phenomenon has reached millions of people overnight and dominated news media for weeks after its release [16,17,18,19]. Health professionals have pointed out potential benefits including increased physical activity, spending more time outside, and exploring the neighborhood and city, social interactions, and mastering game challenges, but have also raised concerns such as injury, abduction, trespassing, violence, and cost [15,33]. In this study, we have precisely quantified the impact of Pokémon Go on physical activity and studied the effect on different groups of individuals.

We found that playing the game significantly increased physical activity on the group-level as well as the individual level over an observation period of approximately 4 weeks. The more interest the users showed in Pokémon Go (measured through intensity of search queries seeking details about game usage), the larger the increase in physical activity. For example, users who issued 10 Pokémon Go queries on details of the game within the 2 months after the release of the game increased their activity by 1479 steps a day or 26%.

We found that Pokémon Go increased the activity all across the studied population, largely independent of prior activity level, age, weight status, or gender. These results are encouraging as they suggest that any positive effects due to Pokémon Go are available even to sedentary, obese, and older users. Effectively reaching these users with physical activity interventions is critical for public health [15].

Comparing Pokémon Go with existing mobile health apps, we found further evidence that Pokémon Go is able to reach low activity populations whereas mobile health and fitness apps largely draw from an already active population. Low activity populations would see the largest benefits from improving their activity [7,8]. This highlights the promise of game-based interventions versus traditional approaches, which have often been ineffective for people with low activity levels [13,14].

Given its great popularity, Pokémon Go has significantly affected US physical activity and added an estimated 144 billion steps to US physical activity, which is about 2724 times around the world or 143 round trips to the moon. Furthermore, highly engaged users were almost 3 times as likely to meet official activity guidelines [7,8,26,27] in the 30 days after starting to play Pokémon Go compared with that before. If this user engagement could be sustained, Pokémon Go would have the potential to measurably affect US life expectancy.

These results emphasize the special contribution that activity-encouraging games could have on physical activity and public health. These games attract a wide range of people including those with low prior physical activity. We have demonstrated that such games can lead to significant activity increases and could have a large impact on the society. However, we have also highlighted challenges in realizing this potential. Most importantly, games would need to be able to sustain long-term engagement and lead to sustained behavior change. Furthermore, these games might not be appealing to everyone (eg, we observed males to be more likely to play the games than females), and clearly these games should not replace but complement existing physical activity programs (eg, [2,9,10,11,12,14]). Understanding how to design games and how to bring together games and health interventions will be important to public health in the future. As a first step, our study helps provide guidance on what could come of continuous engagement and with additional engagement.

### Limitations

Our study is not without limitations. First, the study population is not a random sample of the US population. Subjects were able to afford a wearable device for activity tracking and willing to share their data for research purposes. Our sample of Pokémon Go users is predominantly male. Furthermore, we use individuals search queries as a proxy for playing Pokémon Go and consider the number of queries as indicating the degree of engagement. However, we find strong evidence that the proxies for usage and engagement are effective. The method identifies a fraction of users that is very similar to independent estimates of Pokémon Go penetration in the United States (see Methods) and we find a strong dose-response relationship between the number of Pokémon Go queries and increased physical activity (see Results). User demographic variables were self-reported. Finally, our follow-up period is currently restricted to 30 days. Future work is needed to study the long-term effectiveness of games such as Pokémon Go to increase physical activity.

### Comparison With Prior Work

Few research studies to date have harnessed data obtained from consumer wearable devices to study influences of the devices on physical activity [34]. However, a number of medical studies have examined accelerometer-defined activity [4,31,35], rather than relying on self-report measures. Studies have found that use of pedometers and activity trackers for self-monitoring can help increase activity [36,37], but other studies have reported mixed results [38]. Beyond enabling self-monitoring, encouraging additional activity through reminders lead to increased activity only for the first week after the intervention and did not lead to any significant changes after 6 weeks in a randomized controlled trial [38].

To encourage healthy behavior change, researchers have studied the design of “exergames” [39,40,41], video games combined with exercise activity, and location-based games where game play progresses through a player’s location [42]. However, no such game has been nearly as popular and widely used as Pokémon Go. Such games have yet to be integrated into physical activity programs, even though one US college recently announced a physical education class based on Pokémon Go [43]. Recently, a study demonstrated that social influence in online social networks leads to significant increases in average physical activity over a period of several months [44].

There is a growing body of work on using large-scale search query logs to identify subjects with particular conditions for research studies, including such efforts as detecting adverse reactions to medications and identifying signals that could help with screening for cancer [20,45]. Other work has studied activity-related posts on social media to better understand the sharing of health behaviors [46,47,48], but has not yet connected such data to ground-truth health behaviors or focused on interventions on a large scale.

To the best of our knowledge, this is first study of the link between the usage of Pokémon Go or similar games on physical activity and health. Also, this is the first effort to combine data from wearable devices with information drawn from search engine queries.

### Conclusions

Novel mobile games that require players to physically move in the real world appear to be an effective complement to traditional physical activity interventions, and they are able to reach millions of engaged users. We studied the effect of Pokémon Go on physical activity through a combination of large-scale wearable sensor data with search engine logs and showed that the game leads to significant increases in physical activity over a period of 30 days, particularly with the engaged users increasing their average activity by 1473 steps a day or 26%. Based on our findings, we estimate that the game has already added an estimated 144 billion steps to US physical activity. If engagement with Pokémon Go could be sustained over the lifetime of its many users, we estimate that the game would add an estimated 2.825 million years of additional lifetime to its US users. However, further studies are needed to investigate potential long-term effects. We see great promise for public health in designing geocentric games like Pokémon Go and in working to sustain users’ engagement with them.

## Abbreviations

BMI: body mass index

## References

[1] Miles L (2007). Physical activity and health. Nutrition Bulletin.

[2] Sparling PB, Owen N, Lambert EV, Haskell WL (2000). Promoting physical activity: the new imperative for public health. Health Educ Res.

[3] World Health Organization.

[4] Althoff T, Sosic R, Hicks JL, King AC, Delp SL, Leskovec J (2016). Quantifying Dose Response Relationships Between Physical Activity and Health Using Propensity Scores.

[5] Lee I, Shiroma EJ, Lobelo F, Puska P, Blair SN, Katzmarzyk PT, Lancet Physical Activity Series Working Group (2012). Effect of physical inactivity on major non-communicable diseases worldwide: an analysis of burden of disease and life expectancy. Lancet.

[6] Ding D, Lawson KD, Kolbe-Alexander TL, Finkelstein EA, Katzmarzyk PT, van MW, Pratt M, Lancet Physical Activity Series 2 Executive Committee (2016). The economic burden of physical inactivity: a global analysis of major non-communicable diseases. Lancet.

[7] Centers for Disease Control and Prevention.

[8] https://health.gov/paguidelines/pdf/paguide.pdf.

[9] Reis RS, Salvo D, Ogilvie D, Lambert EV, Goenka S, Brownson RC, Lancet Physical Activity Series 2 Executive Committee (2016). Scaling up physical activity interventions worldwide: stepping up to larger and smarter approaches to get people moving. Lancet.

[10] Sallis JF, Bauman A, Pratt M (1998). Environmental and policy interventions to promote physical activity. Am J Prev Med.

[11] Dobbins M, DeCorby K, Robeson P, Husson H, Tirilis D (2009). School-based physical activity programs for promoting physical activity and fitness in children and adolescents aged 6-18. Cochrane Database Syst Rev.

[12] Salmon J, Booth ML, Phongsavan P, Murphy N, Timperio A (2007). Promoting physical activity participation among children and adolescents. Epidemiol Rev.

[13] Dishman RK, Sallis JF, Orenstein DR (1985). The determinants of physical activity and exercise. Public Health Rep.

[14] Marshall AL (2004). Challenges and opportunities for promoting physical activity in the workplace. J Sci Med Sport.

[15] American Heart Assocation.

[16] SurveyMonkey Intelligence Blog.

[17] Wong J https://www.theguardian.com/technology/2016/aug/23/pokemon-go-active-users-down-augmented-reality-games.

[18] Lynley M TechCrunch.

[19] Perez S https://techcrunch.com/2016/07/13/pokemon-go-tops-twitters-daily-users-sees-more-engagement-than-facebook/.

[20] Paparrizos J, White RW, Horvitz E (2016). Screening for Pancreatic Adenocarcinoma Using Signals From Web Search Logs: Feasibility Study and Results. J Oncol Pract.

[21] White RW, Wang S, Pant A, Harpaz R, Shukla P, Sun W, DuMouchel W, Horvitz E (2016). Early identification of adverse drug reactions from search log data. J Biomed Inform.

[22] Tucker JM, Welk GJ, Beyler NK (2011). Physical activity in U.S. Adults: Compliance with the Physical Activity Guidelines for Americans. Am J Prev Med.

[23] Efron B, Tibshirani RJ (1994). An introduction to the bootstrap.

[24] Dunn AL, Trivedi MH, O'Neal HA (2001). Physical activity dose-response effects on outcomes of depression and anxiety. Med Sci Sports Exerc.

[25] Lee IM, Skerrett PJ (2001). Physical activity and all-cause mortality: what is the dose-response relation?. Med Sci Sports Exerc.

[26] Tudor-Locke C, Craig CL, Brown WJ, Clemes SA, De CK, Giles-Corti B, Hatano Y, Inoue S, Matsudo SM, Mutrie N, Oppert J, Rowe DA, Schmidt MD, Schofield GM, Spence JC, Teixeira PJ, Tully MA, Blair SN (2011). How many steps/day are enough? For adults. Int J Behav Nutr Phys Act.

[27] Tudor-Locke C, Leonardi C, Johnson WD, Katzmarzyk PT, Church TS (2011). Accelerometer steps/day translation of moderate-to-vigorous activity. Prev Med.

[28] Dwyer T, Pezic A, Sun C, Cochrane J, Venn A, Srikanth V, Jones G, Shook RP, Shook R, Sui X, Ortaglia A, Blair S, Ponsonby A (2015). Objectively Measured Daily Steps and Subsequent Long Term All-Cause Mortality: The Tasped Prospective Cohort Study. PLoS One.

[29] United States Social Security Administration.

[30] National Institute of Diabetes and Digestive and Kidney Diseases.

[31] Tudor-Locke C, Bassett DR Jr (2004). How many steps/day are enough? Preliminary pedometer indices for public health. Sports Med.

[32] Dishman RK, Buckworth J (1996). Increasing physical activity: a quantitative synthesis. Med Sci Sports Exerc.

[33] Serino M, Cordrey K, McLaughlin L, Milanaik RL (2016). Pokémon Go and augmented virtual reality games: a cautionary commentary for parents and pediatricians. Curr Opin Pediatr.

[34] Hayden EC (2016). Mobile-phone health apps deliver data bounty. Nature.

[35] Troiano RP, Berrigan D, Dodd KW, Mâsse LC, Tilert T, McDowell M (2008). Physical activity in the United States measured by accelerometer. Med Sci Sports Exerc.

[36] Thorup C, Hansen J, Grønkjær M, Andreasen JJ, Nielsen G, Sørensen EE, Dinesen BI (2016). Cardiac Patients' Walking Activity Determined by a Step Counter in Cardiac Telerehabilitation: Data From the Intervention Arm of a Randomized Controlled Trial. J Med Internet Res.

[37] Wang JB, Cataldo JK, Ayala GX, Natarajan L, Cadmus-Bertram LA, White MM, Madanat H, Nichols JF, Pierce JP (2016). Mobile and Wearable Device Features that Matter in Promoting Physical Activity. J Mob Technol Med.

[38] Wang JB, Cadmus-Bertram LA, Natarajan L, White MM, Madanat H, Nichols JF, Ayala GX, Pierce JP (2015). Wearable Sensor/Device (Fitbit One) and SMS Text-Messaging Prompts to Increase Physical Activity in Overweight and Obese Adults: A Randomized Controlled Trial. Telemedicine and e-Health.

[39] Göbel S, Hardy S, Wendel V, Mehm F, Steinmetz R (2010). Serious games for health: Personalized exergames.

[40] Sinclair J, Hingston P, Masek M (2007). Considerations for the design of exergames.

[41] Staiano AE, Calvert SL (2011). Exergames for Physical Education Courses: Physical, Social, and Cognitive Benefits. Child Dev Perspect.

[42] Avouris NM, Yiannoutsou N (2012). A review of mobile location-based games for learning across physical and virtual spaces. J Univers Comput Sci.

[43] Ryssdal K (2016). A college is offering a Pokémon Go class. Marketplace..

[44] Althoff T, Jindal P, Leskovec J (2017). Online Actions with Offline Impact: How Online Social Networks Influence Online and Offline User Behavior.

[45] White RW, Horvitz E (2014). From health search to healthcare: explorations of intention and utilization via query logs and user surveys. J Am Med Inform Assoc.

[46] Kendall L, Hartzler A, Klasnja P, Pratt W (2011). Descriptive analysis of physical activity conversations on Twitter.

[47] Park K, Weber I, Cha M, Lee C (2016). Persistent sharing of fitness app status on Twitter.

[48] Teodoro R, Naaman M (2013). Fitter with Twitter: Understanding personal health and fitness activity in social media.

